# Localization of Nerve Growth Factor Expression to Structurally Damaged Cartilaginous Tissues in Human Lumbar Facet Joint Osteoarthritis

**DOI:** 10.3389/fimmu.2022.783076

**Published:** 2022-03-01

**Authors:** Matthias F. Seidel, Cordula Netzer, Véronique Chobaz, Thomas Hügle, Jeroen Geurts

**Affiliations:** ^1^ Department of Rheumatology, Spitalzentrum-Centre Hospitalier, Biel-Bienne, Switzerland; ^2^ Spine Surgery, Department of Biomedical Engineering, University Hospital of Basel, Basel, Switzerland; ^3^ Rheumatology, Department of Musculoskeletal Medicine, Lausanne University Hospital, Lausanne, Switzerland

**Keywords:** nerve growth factor, osteoarthritis, facet joint, lumbar spine, spinal stenosis

## Abstract

**Purpose:**

Nerve Growth Factor (NGF) is a pivotal mediator of chronic pain and plays a role in bone remodelling. Through its high affinity receptor TrkA, NGF induces substance P (SP) as key downstream mediator of pain and local inflammation. Here we analysed NGF, TrkA and SP tissue distribution in facet joint osteoarthritis (FJOA), a major cause of chronic low back pain.

**Methods:**

FJOA specimens (*n=*19) were harvested from patients undergoing intervertebral fusion surgery. Radiologic grading of FJOA and spinal stenosis, followed by immunohistochemistry for NGF, TrkA and SP on consecutive tissue sections, was performed in ten specimens. Explant cultures (*n*=9) were used to assess secretion of NGF, IL-6, and SP by FJOA osteochondral tissues under basal and inflammatory conditions.

**Results:**

NGF was predominantly expressed in damaged cartilaginous tissues (80%), occasionally in bone marrow (20%), but not in osteochondral vascular channels. NGF area fraction in cartilage was not associated with the extent of proteoglycan loss or radiologic FJOA severity. Consecutive sections showed that NGF and SP expression was localized at structurally damaged cartilage, in absence of TrkA expression. SP and TrkA were expressed in subchondral bone marrow in both presence and absence of NGF. Low level NGF, but not SP secretion, was detected in four out of eighteen FJOA explants under both basal or inflammatory conditions (*n*=2 each).

**Conclusion:**

NGF is associated with SP expression and structural cartilage damage in osteoarthritic facet joints, but not with radiologic disease severity. NGF tissue distribution in FJOA differs from predominant subchondral bone expression reported for knee OA.

## Introduction

Nerve growth factor (NGF) is an important mediator in chronic pain conditions and is upregulated in osteoarthritis (OA) and other rheumatic disorders (1). NGF can bind to high (TrkA) and low affinity (p75) receptors and signalling through TrkA induces expression of substance P (SP), which links action potentials from pain fibres to the spinal cord (2). In addition, SP is released from nociceptors after antidromic transport and mediates local inflammatory events (3). NGF expression in human knee OA has been primarily found in osteochondral vascular channels in subchondral bone and correlated with symptomatic chondropathy, but not disease severity (4). In addition, both NGF and TrkA were found expressed in isolated chondrocytes from intact and damaged knee cartilage, demonstrating increased expression in the latter (5).

Human facet joint osteoarthritis (FJOA) is a major cause of chronic low back pain (CLBP). Increased expression of NGF and TrkA in FJOA capsular tissues has been described to correlate with gross morphological assessment of facet joint degeneration (6). Similarly, increased NGF expression in the capsular ligament was found in an experimental model of painful facet joint distraction (7). Whether NGF expression and signalling occurs in additional FJOA tissues, such as articular cartilage and subchondral bone marrow, remains unknown.

Specific NGF antagonists (NGFi) are an emerging class of pain medication with clinical efficacies in patients with OA and to some extent with CLBP [reviewed in (8)]. One of the most serious adverse events in NGFi clinical trials is rapidly progressive OA (RPOA) of peripheral joints. RPOA type 1 is defined as rapid loss of joint space width within approximately 1 year without evidence of bone loss or destruction whilst type 2 is associated with progressive bone destruction (9). Recent studies revealed efficacy of NGFi in improving pain and physical function in knee OA, while RPOA remained a serious adverse event in 1-3% of the treated patients (10). In a cohort of chronic CLBP patients NGFi demonstrated efficacious in reducing CLBP intensity and RPOA was almost exclusively observed in individuals with additional peripheral OA (11).

The objective of this study was to describe the tissue distribution and secretion of key molecules of the NGF signalling axis in cartilage and subchondral bone tissue compartments in FJOA. In addition, we investigated whether NGF tissue expression was associated with radiological severity of FJOA and lumbar spinal stenosis.

## Materials and Methods

### Patient Characteristics

The study protocol has been reviewed and approved by the Ethics Committee of Northwestern Switzerland (Number 147/12). Written consent was obtained from all patients. Facet joint specimens were obtained from patients suffering from symptomatic stenosis of the lumbar spine for >6 months. These patients underwent single-level decompression and fusion surgery (L1-S1 levels). In all cases the routine transforaminal lumbar intercorporal fusion (TLIF) technique was applied. In all nineteen patients (70 ± 14 years, 11 female) the processus articularis superior of the dissected facet joint (T10 *n*=1, L2 *n*=2, L3 *n*=3, L4 *n=*8, L5 *n*=5) was collected and transported in saline solution for immediate processing for histology (*n*=10, 5 female) or explant cultures (*n*=9, 6 female). All patients reported pain radiating down at least one leg and self-assessed walking distance varied between 20-1000 meters. Pain medication included paracetamol (*n*=1), metamizole (*n*=1), conventional NSAIDs (*n*=4), opioids (*n*=4), or none (*n*=9).

### Magnetic Resonance Imaging

MRI of the lumbar spine was performed on a 1.5-T scanner using a standard protocol comprising, T1-, T2- and fat-saturated T2-weighted sequences. MRI images were evaluated twice by a senior spine consultant with 15 years of experience in reading MRI of the spine. FJOA was graded T1- and T2-weighted sequences in sagittal and axial planes using the Weishaupt grading system (12). Grade 1 = joint space narrowing (JSN) and/or small osteophytes and/or mild hypertrophy of articular processes. Grade 2 = JSN and/or moderate osteophytes and/or moderate hypertrophy of the articular process and/or mild subarticular bone erosions. Grade 3 = JSN and/or large osteophytes and/or severe hypertrophy of the articular process and/or severe subarticular bone erosions and/or subchondral cysts. Synovitis was defined as T2 hyperintensity in and adjacent to facet joints in axial fat-saturated T2-weighted images. Severity of lumbar spinal stenosis was graded on T2-weigthed axial images according to Schizas (13), grade A = no or minor stenosis, B = moderate stenosis, C = severe stenosis and D = extreme stenosis.

### Histological Tissue Processing and Analysis

The medial portion of the processus articularis superior comprising the facet joint and capsular tissue (*n*=10, 68 ± 16 years, 5 female) was collected by partial facetectomy and formalin-fixed at 4°C for 2 days. Specimens were decalcified in 5% formic acid for 5-7 days, rinsed in PBS twice for 1 hour and embedded in paraffin. Cartilage degeneration and proteoglycan loss was assessed on 6 μm sagittal sections stained with Safranin-*O/*Fast Green. Quantitative assessment of Safranin-*O* staining was performed using ImageJ (version 1.53c, National Institutes of Health, USA) by measuring area fraction on tresholded images after color deconvolution (Masson/Trichrome filter). Regions of interest (ROIs) were placed on cartilage tissue only.

### Immunohistochemistry

After deparaffinization, tissue sections were subsequently incubated in 3% H_2_O_2_ for 10 minutes and antibody diluent for IHC for 45 minutes (Catalogue no. 559148, BD Pharmingen, Eysins, Switzerland) to block endogenous peroxidase activity and non-specific antibody binding, respectively. Consecutive 6 μm tissue sections were incubated overnight at 4°C in antibody diluent with the following antibodies: monoclonal mouse anti-TrkA (Clone OTI5B6, dilution 1:150, Novus Biologicals, Centennial, CO, USA), monoclonal rabbit anti-NGF (Clone EP1320Y, dilution 1:250, Abcam, Cambridge, UK), mouse monoclonal anti-SP (Clone 266815, dilution 1:50, Novus Biologicals). Macrophage staining was performed after heat-mediated antigen retrieval (10 mM citrate buffer pH 6.0 at 70°C for 25 min) using monoclonal mouse anti-CD68 (Clone KP1, dilution 1:100, Zytomed Systems, Berlin, Germany) for 1 hour at RT. Immunoreactivities were visualized using mouse- and rabbit-specific peroxidase kits according to the manufacturer’s instructions (Vectastain Elite ABC kit, Vector Laboratories, Burlingame, CA, USA). Negative controls were performed with isotype control antibodies (**Supplementary Table** and **Supplementary Figure 1**). Stained tissue sections were counterstained with Papanicolaou (Merck AG, Dietikon, Switzerland). NGF area fraction in cartilage tissue was quantified on tresholded images after color deconvolution (Hematoxylin/DAB filter) using ImageJ.

### Explant Culture of Facet Joints and ELISA Measurements

Dissected facet joint specimens (*n=*9, 72 ± 12 years, 6 female) were processed aseptically and cut in two equal-sized coronal 2 mm samples using a scalpel and cultured as previously described (14). Briefly, specimens were cultured for 7 days at 37˚C in 8 mL αMEM medium (10% fetal bovine serum, 10 mM HEPES, 4 mM L-glutamine, 50 μM L-ascorbic acid-2-phosphate and 10 mM sodium β-glycerophosphate pentahydrate, 10^-7^ M dexamethasone) in the presence and absence of 1 μg/mL lipopolysaccharide from *Escherichia coli* O111:B4 (Sigma-Aldrich, Buochs, Switzerland). LPS treatment was added on day 1 and 4. Supernatants were collected, centrifuged, aliquoted and stored at -80˚C until ELISA measurement. Concentrations of human interleukin-6 (IL-6), NGF and SP were determined in the supernatants using ELISA kits (Abcam ab178013, ab99986 and ab133029, respectively). Secreted protein levels were normalized for wet tissue weight and expressed as pg protein/mg tissue.

### Statistical Analysis

Statistical analyses were performed using GraphPad Prism (v6.2, Graphpad Software Inc., San Diego, CA, USA). Data following normal distribution were reported as means ± SD and non-parametric data as median with interquartile range. Significant differences between treatment groups were calculated using ratio paired *t*-test. Association between quantitative histological assessments and MRI-based severity grades were determined using Spearman’s rank correlation. *P*-values less than 0.05 were considered significant.

## Results

### NGF Is Predominantly Localized to Damaged Cartilaginous Tissues

**Figure 1:**
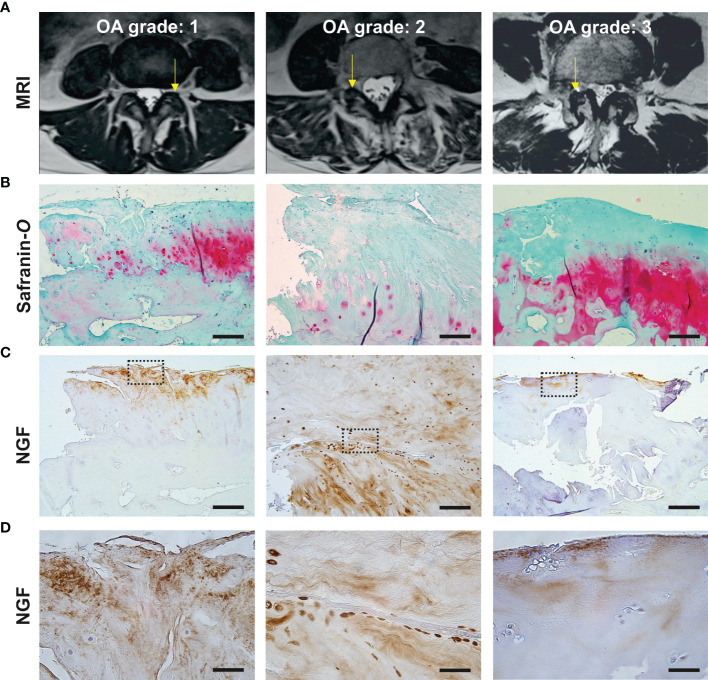
NGF expression localizes to damaged cartilaginous tissues in facet joint osteoarthritis. **(A)** Representative axial T2 MRI images illustrating Weishaupt grades 1-3 of FJOA severity. The affected facet joint is indicated with an arrow. **(B, C)** Consecutive tissue sections of dissected facet joints indicated on MRI images above, stained with Safranin-*O* (proteoglycans, red) or anti-NGF (brown). Scale bar = 500 μm **(D)** High magnification view of NGF staining patterns in the marked area in images above. Scale bar = 50 μm.

**Figure 2:**
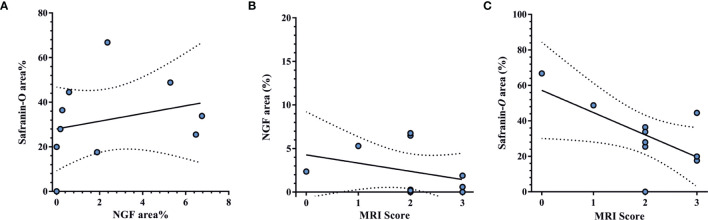
Lack of correlation between NGF expression and OA severity. **(A)** Distribution of NGF area fraction relative to Safranin-*O* area fraction in cartilaginous tissues. **(B)** Distribution of NGF area fraction relative to MRI-assessed OA severity. **(C)** Distribution of Safranin-*O* area fraction in cartilaginous tissue relative to MRI-assessed OA severity Regression analysis (solid line) with 95% confidence intervals (dotted line) are depicted.

Lumbar facet joint specimens were obtained from patients with moderate to severe radiologic OA (**Figure 1A**), indicated by a median Weishaupt grade of 2 [IQR: 1.75–3] (**Table 1**). Safranin-*O*-stained tissue sections displayed hallmarks of severe cartilage degeneration including surface damage, fissures and loss of proteoglycan content (**Figure 1B**). NGF expression was detected in nine out of ten FJOA specimens, predominantly in cartilage (*n*=8) and occasionally in marrow tissue (*n*=2) (**Table 1**). Microscopic analysis of NGF expression and proteoglycan distribution in consecutive tissue sections revealed that NGF localized almost exclusively to damaged regions of cartilaginous tissue (**Figure 1C**). High magnification views showed NGF localization in the extracellular matrix as well as pericellular chondrocyte staining (**Figure 1D**). Correlation analyses demonstrated that NGF area fraction did not significantly correlate with the extent of proteoglycan loss (*r*=0.25, *p*=0.50, **Figure 2A**) or Weishaupt grade (*r*= –0.46, *p*=0.15, **Figure 2B**). Radiologic OA severity was negatively correlated with the cartilage proteoglycan content in Safranin-*O*-stained cartilaginous tissue (*r*= –0.69, *p*=0.02, **Figure 2C**). Presence of synovitis was positively correlated with NGF area fraction (*r*= 0.78, *p*= 0.03), but not with radiological severity (*r*= –0.09, *p*= 0.38) or Safranin-*O* area fraction (*r*= –0.26, *p*= 0.28).

**Table 1:** Patient demographics, radiological scores and NGF tissue distribution.

Age	Gender (M/F)	Spine Level	Weishaupt grade (0-3)	Synovitis (Yes/No)	Schizas score (A-D)	NGF cartilage (Yes/No)	NGF marrow (Yes/No)
32	F	L5 - S1	0	Yes	D	Yes	No
78	M	L2 - L3	2	No	B	Yes	No
76	M	L3 - L4	2	No	B	Yes	No
61	M	L4 - L5	2	No	B	Yes	No
60	F	L5 - S1	2	No	B	No	No
82	F	L3 - L4	3	Yes	D	Yes	Yes
80	M	L3 - L4	3	Yes	D	Yes	No
71	F	L4 - L5	1	Yes	A	Yes	No
82	M	L3 - L4	3	Yes	C	Yes	No
57	F	L4 - L5	3	No	C	No	Yes

### NGF Localized With SP, but Not TrkA Expression in FJOA

**Figure 3:**
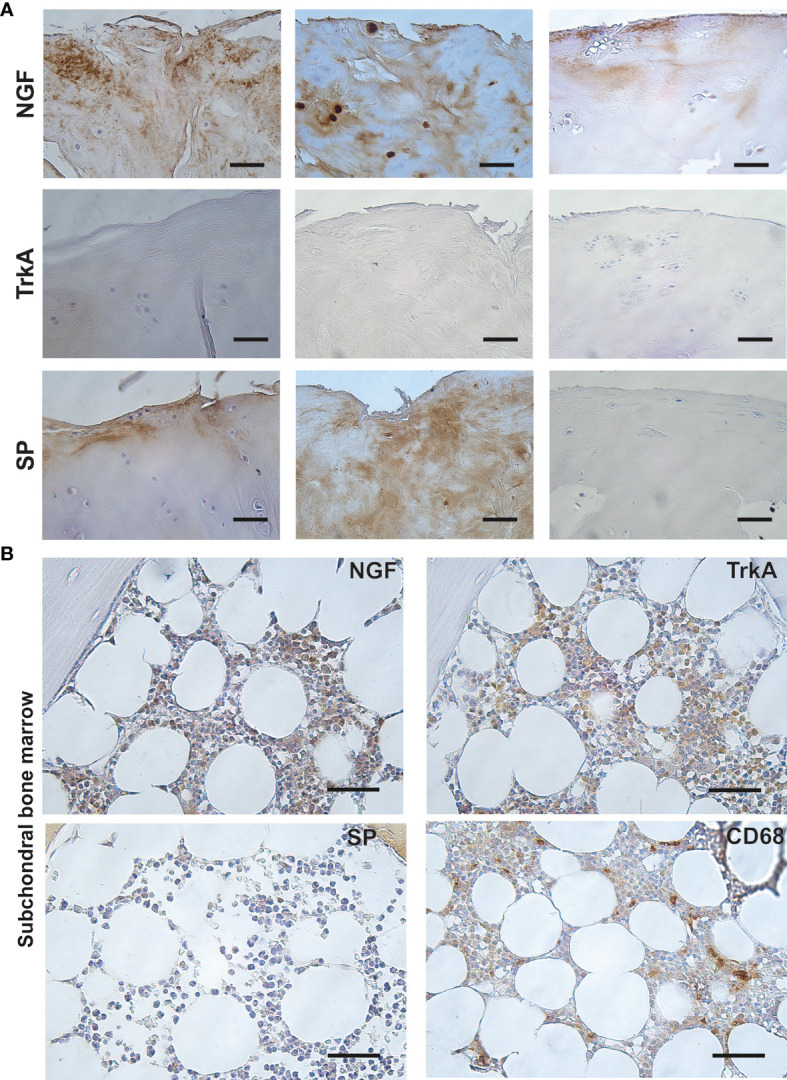
Localization of NGF, SP and TrkA in facet joint osteoarthritis. **(A)** Representative images of consecutive tissue sections stained for NGF, TrkA and SP, revealing localization of NGF and SP, but not TrkA in damaged regions of cartilaginous tissue. Scale bar = 50 μm. **(B)** Consecutive tissue sections showing distribution of NGF, TrkA, SP and CD68 staining in subchondral bone marrow tissue. Scale bar = 50 μm.

Next, we analysed distribution of NGF, its high affinity receptor TrkA and downstream neuropeptide SP in consecutive tissue sections. TrkA expression was detected in neither chondrocytes, nor extracellular matrix of cartilaginous tissues. SP was expressed in seven out of eight specimens displaying cartilaginous NGF expression. Extracellular staining patterns for SP and NGF were largely overlapping in consecutive tissue sections (**Figure 3A**). In contrast to cartilaginous tissue, TrkA and SP displayed respectively abundant and focal expression in subchondral bone marrow cells in eight out of ten specimens. CD68+ macrophages showed a focal expression pattern in all specimens. Specimens displaying NGF expression in bone marrow (*n*=2) revealed abundant cellular expression (**Figure 3B**).

### Facet Joint Explants Secrete Low Levels of NGF, but Not SP

**Figure 4:**
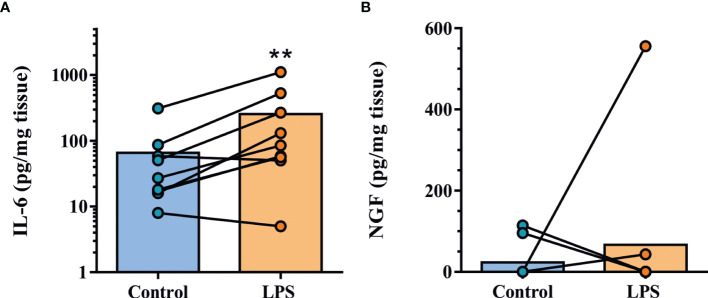
IL-6 and NGF secretion by human facet joint explant cultures. **(A)** Assessment of IL-6 tissue secretion in explants from nine patients under untreated (control) or inflammatory conditions (LPS). **(B)** NGF tissue secretion was detected in four patient specimens. Lines identify paired samples from patients. **P < 0.01 by ratio paired *t*-test.

Finally, we sought to determine whether NGF and SP are secreted by FJOA specimens under basal and inflammatory conditions. For this, fresh osteochondral explants were left either untreated or stimulated with 1 μg/mL LPS (*n*=9 patients, *n=*18 explants). This treatment activates tissue macrophages and mimicks inflammatory signalling induced by damage-associated molecular patterns in joint tissues (15). As a positive control, LPS challenge led to four-fold elevated tissue secretion of IL-6 compared with untreated specimens (**Figure 4A**). Low level NGF secretion was detected in explants from four patients (*n*=2 untreated, *n*=2 LPS-treated) (**Figure 4B**). In contrast, SP was not detected in FJOA explant-conditioned medium (data not shown).

## Discussion

Here we identified predominant NGF and SP expression in damaged cartilaginous tissue of osteoarthritic lumbar facet joints from patients with spinal stenosis. Analogous to human knee OA (4), NGF expression was not correlated with OA severity or synovitis grade. However, NGF tissue distribution differed considerably showing scarce expression in facet joint subchondral bone, whereas the osteochondral junction was the major tissue expressing NGF in osteoarthritic knee joints.

The contrasting NGF tissue distribution between facet joint and knee OA might provide insight into the frequency of RPOA as adverse events in NGFi treatment. Recent studies have demonstrated a dose-dependent increase of RPOA in patients with knee OA or CLBP treated with NGFi (10, 11), yet this rarely occurred in CLBP patients without peripheral OA. NGF has been described as a regulator of osteogenesis and bone turnover (16), however, scarce expression in bone marrow would argue against a significant contribution of NGF signalling to FJOA bone remodelling. Instead, predominant NGF expression in damaged cartilaginous tissues provides a rationale for improving pain in FJOA-related CLBP.

Several studies have documented cartilaginous NGF gene expression in human or experimental OA (5, 17–20). Pain-sensitizing genes, including NGF, were found to be upregulated by mechanical injury in murine knee joints in a transforming growth factor-beta-dependent fashion (18). The same factor was implied in mediating non-inflammatory NGF expression in human cartilage tissue and isolated chondrocytes (17). *In vitro* treatment of cartilage with NGF led to increased proteoglycan secretion and upregulation of matrix-degrading enzymes (21), while isolated chondrocytes displayed increased mineralization under pro-osteogenic conditions (20). Together, these studies suggest elevated NGF expression is common in degenerated cartilage and may promote cartilage matrix catabolism.

There are a number of limitations pertaining to the methodology of this study. This cross-sectional study using a small sample size of primarily end-stage FJOA samples from lumbar spinal stenosis patients revealed abundant NGF expression in damaged cartilaginous tissue. However, the results may not predict which subgroup of CBLP patients might respond to NGFi treatment. Furthermore, histological studies of FJOA specimens from patients with and without RPOA upon NGFi treatment would be required to assess whether differential NGF tissue distribution, in subchondral bone marrow for instance, associates with RPOA. Evaluation of radiological imaging was performed by a single reader and we acknowledge caution has to be exerted when interpreting protein expression at the cartilage tissue level with severity scores based on whole joint morphology. Lastly, immunohistochemistry analyses using consecutive tissue sections allowed for localizing NGF, TrkA and SP to similar regions, yet would ideally utilize immunofluorescence microscopy of double-stained tissues to determine the extent of co-localization.

We have previously described the presence of CD68-positive macrophages in subchondral marrow tissues of FJOA specimens (22). In knee OA, both CD68-positive macrophages and NGF-positive cells in part co-localized in areas of inflammation of the synovial lining and sublining regions suggesting that macrophages also express NGF (23). Co-localization analyses may aid in identifying the cellular source of NGF in subchondral bone marrow. Whether macrophages and/or NGF expression associate with pain in FJOA remains to be elucidated.

In summary, NGF tissue distribution in human FJOA shows obvious differences as compared to knee OA. The lack of NGF expression in facet joint bone marrow tissue might provide a clue for different frequencies of incident RPOA upon NGFi treatment in patients with knee or facet joint OA. Further studies are needed to establish NGFi as safe and efficient treatment options for CLBP and other chronic pain conditions such as tumor pain or chronic regional pain syndrome. NGF might thus be a promising treatment target molecule in CLBP.

## Data Availability Statement

The raw data supporting the conclusions of this article will be made available by the authors, without undue reservation.

## Ethics Statement

The studies involving human participants were reviewed and approved by Ethics Committee of Northwestern and Central Switzerland. The patients/participants provided their written informed consent to participate in this study.

## Author Contributions

All authors contributed to the study conception and study design. Material preparation, data collection and analysis were performed by MS, CN, VC, TH, and JG. The first draft of the manuscript was written by MS, TH, and JG. All authors read and approved the final manuscript.

## Funding

This work was supported by intramural funds from the Department of Rheumatology, Lausanne University Hospital (CHUV) and the Department of Rheumatology, Spitalzentrum - Centre hospitalier Biel-Bienne.

## Conflict of Interest

MS has received advisory board honoraria from Pfizer & Eli Lilly.

The remaining authors declare that the research was conducted in the absence of any commercial or financial relationships that could be construed as a potential conflict of interest.

## Publisher’s Note

All claims expressed in this article are solely those of the authors and do not necessarily represent those of their affiliated organizations, or those of the publisher, the editors and the reviewers. Any product that may be evaluated in this article, or claim that may be made by its manufacturer, is not guaranteed or endorsed by the publisher.
