# Supplementary material for: Localization of Nerve Growth Factor Expression to Structurally Damaged Cartilaginous Tissues in Human Lumbar Facet Joint Osteoarthritis
Source: Front Immunol. 2022 Mar 1;13:783076. doi: 10.3389/fimmu.2022.783076 (PMC8921992; doi:10.3389/fimmu.2022.783076)
Supplement: Supplementary file 1 [file DataSheet_1.docx]

Supplementary Material

**Supplementary Table 1**

Primary antibodies and isotype controls used for immunohistochemistry on formalin-fixed and formic acid decalcified human facet joint specimens.

| **Clone** | **Isotype** | **Antigen** | **Host** | **Dilution** | **Retrieval** | **Supplier** | **Reference** |
| --- | --- | --- | --- | --- | --- | --- | --- |
| OTI5B6 | IgG1 | TrkA | mouse | 1:200 | none | Novus Biologicals | [[1](#_ENREF_1)] |
| EP1320Y | IgG | NGF | rabbit | 1:100 | none | Abcam | [[2](#_ENREF_2)] |
| 266815 | IgG1 | SP | mouse | 1:100 | none | Novus Biologicals | [[3](#_ENREF_3)] |
| KP1 | IgG1 | CD68 | mouse | 1:100 | heat-induced | Zytomed Systems | [[4](#_ENREF_4)] |
| 11711 | IgG1 | Isotype | mouse | 1:100 | none | R&D Systems | [[5](#_ENREF_5)] |
| EPR25A | IgG | Isotype | rabbit | 1:100 | none | Abcam | [[6](#_ENREF_6)] |

**References**

1. Choi Y, Won YJ, Lee S, Kim A, Kim Y, Park WY, Jo HJ, Song GA, Kwon CH, Park DY (2018) Cytoplasmic TrkA Expression as a Screen for Detecting NTRK1 Fusions in Colorectal Cancer. Transl Oncol 11, 764-770, 10.1016/j.tranon.2018.03.011.

2. Binch A, Snuggs J, Le Maitre CL (2020) Immunohistochemical analysis of protein expression in formalin fixed paraffin embedded human intervertebral disc tissues. JOR Spine 3, e1098, 10.1002/jsp2.1098.

3. Okamura Y, Mishima S, Kashiwakura JI, Sasaki-Sakamoto T, Toyoshima S, Kuroda K, Saito S, Tokuhashi Y, Okayama Y (2017) The dual regulation of substance P-mediated inflammation via human synovial mast cells in rheumatoid arthritis. Allergol Int 66S, S9-S20, 10.1016/j.alit.2017.03.002.

4. McGinley LM, Willsey MS, Kashlan ON, Chen KS, Hayes JM, Bergin IL, Mason SN, Stebbins AW, Kwentus JF, Pacut C, Kollmer J, Sakowski SA, Bell CB, 3rd, Chestek CA, Murphy GG, Patil PG, Feldman EL (2021) Magnetic resonance imaging of human neural stem cells in rodent and primate brain. Stem Cells Transl Med 10, 83-97, 10.1002/sctm.20-0126.

5. Mah V, Elshimali Y, Chu A, Moatamed NA, Uzzell JP, Tsui J, Schettler S, Shakeri H, Wadehra M (2021) ALDH1 expression predicts progression of premalignant lesions to cancer in Type I endometrial carcinomas. Sci Rep 11, 11949, 10.1038/s41598-021-90570-3.

6. Radhakrishnan R, Kowluru RA (2021) Long Noncoding RNA MALAT1 and Regulation of the Antioxidant Defense System in Diabetic Retinopathy. Diabetes 70, 227-239, 10.2337/db20-0375.

**
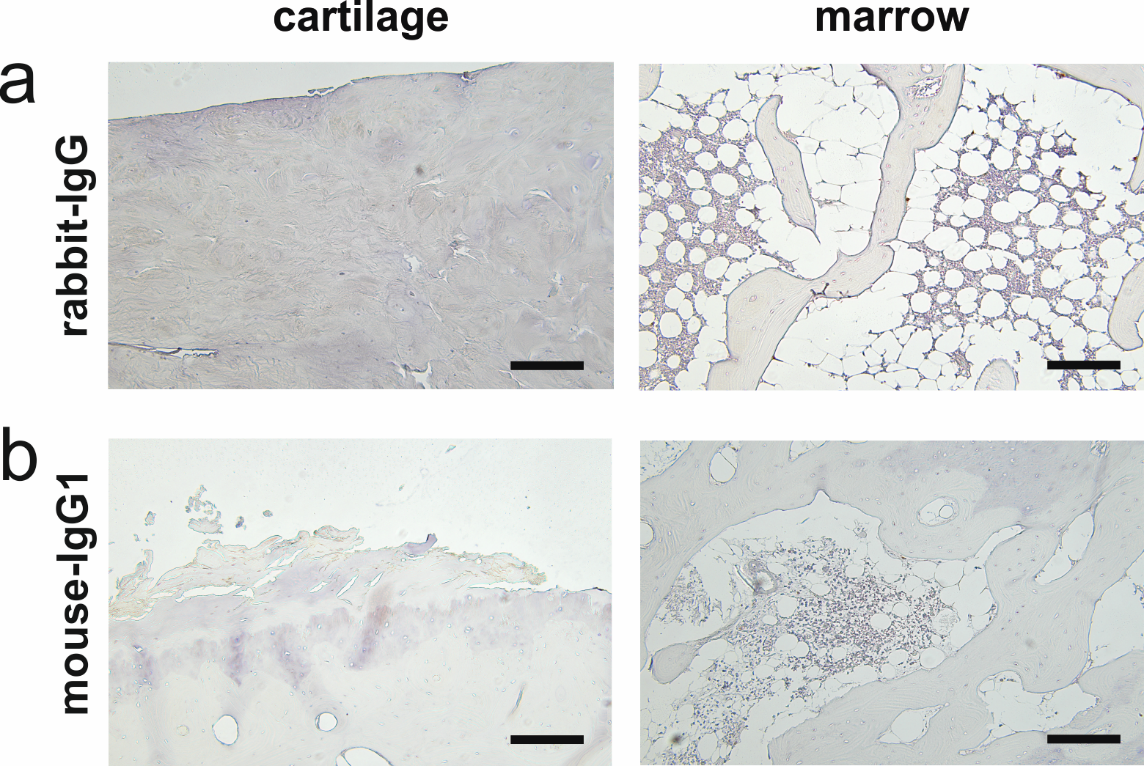
**

**Supplementary Figure 1: Isotype control staining of cartilage and marrow tissues of human facet joints.** (**A**) Isotype control (rabbit IgG) for anti-NGF staining. (**B**) Isotype control (mouse IgG1) for anti-TrkA, Substance P and CD68 staining. Isotype controls did not reveal cellular or pericellular matrix staining in cartilage or bone marrow tissue. Scale bar = 500 μm.
